# Toward predictive prosthetic socket modeling: Preliminary linear regression models of transradial limb–socket geometry

**DOI:** 10.1371/journal.pdig.0001767

**Published:** 2026-10-05

**Authors:** Vishal Pendse, Nader Allam, Calvin C. Ngan, Elaine Lorette, Isabelle Morin-Girard, Jan Andrysek

**Affiliations:** 1 Institute of Biomedical Engineering, University of Toronto, Toronto, Ontario, Canada; 2 Holland Bloorview Kids Rehabilitation Hospital, Toronto, Ontario, Canada; University of Pennsylvania, UNITED STATES OF AMERICA

## Abstract

Socket fit is essential for successful upper-limb prosthetic devices, yet current design workflows remain labor-intensive and dependent on clinician experience. Several digital and algorithmic approaches have been proposed to partially automate socket design, many of which rely on characterizing conventional design practice. In upper-limb prosthetics, prior research has quantified transradial socket rectification practices, but the direct geometric relationship between the residual limb and final socket remains unclear despite its importance to digital socket design workflows. This study examined whether simple linear regression models could provide a preliminary, interpretable description of limb–socket geometric relationships in transradial socket design. Fourteen participants with transradial limb absence were included, whose digitized limb–socket pairs were aligned using a novel spline-based method. Relationships were quantified using global descriptors (volume, surface area, and proximal–distal length) and cross-sectional descriptors at 25%, 50%, and 75% of limb length (mediolateral length, anterior–posterior length, circumference, and cross-sectional area). For each descriptor, simple linear regressions were fit to predict socket geometry from limb geometry, and model performance was evaluated using leave-one-out cross-validation. Global descriptors demonstrated strong linear relationships, with R² values ranging from 0.94 to 0.97 and predicted R² values from 0.92 to 0.97, indicating that overall socket size is largely predictable from limb size. Cross-sectional descriptors showed more variable performance, with the strongest results at mid-length and weaker relationships toward the proximal and distal ends. Mean absolute percentage error ranged from 2.5% to 13.6% across descriptors, while retention values were generally high, indicating limited overfitting. These findings suggest that simple linear models can capture broad scaling relationships between transradial limbs and sockets, but are less able to explain localized geometry in regions where trimline design, suspension strategy, and distal-end clearance play a greater role.

## Author summary

Designing a comfortable and functional prosthetic socket for upper-limb absence is a skilled and time-intensive process, with the overall success of the prosthesis being highly dependent on the fit of the socket. Although digital tools are increasingly used in prosthetic care, many important design steps still depend on clinician experience and are difficult to translate into software. One challenge is that the geometric relationship between a person’s residual limb and the final socket shape is not well understood. In this study, we examined whether simple linear regression models could describe how the shape of the residual limb relates to that of the prosthetic socket. Using 3D scans of limb–socket pairs from 14 people with transradial limb absence, we compared their global and cross-sectional geometric features. We found that overall socket size was strongly related to overall limb size, meaning that broad scaling patterns can be predicted reasonably well. However, local socket geometry was harder to predict near both ends of the model, where design features such as trimlines, suspension, and distal clearance play a larger role. These findings provide an early quantitative foundation for data-driven prosthetic socket design and may help support future digital tools that complement clinical expertise.

## 1. Introduction

The design and provision of custom-fitted prosthetic sockets remain time-intensive, largely manual, and depend on clinician expertise [1]. As the interface between a user’s residual limb and the prosthesis, the socket affects the stability, control, comfort, and load transfer during daily activities [2]. Broadly, socket design involves three main steps: shape capture of the residual limb, rectification, and fabrication [3]. In conventional practice, these steps are typically performed through plaster casting, manual rectification (which involves modifying the shape of the plaster model to reach the desired socket shape), and socket fabrication. In digital workflows, they are instead carried out using optical scanning, software-based rectification, and digital fabrication methods such as additive manufacturing [4–6].

Rectification is a critical stage in socket design, where the captured residual-limb shape is intentionally modified to create a socket shape with appropriate fit and mechanical functionality [2,7,8]. In practice, rectification includes the selective compression of soft tissue, creating reliefs over sensitive or bony regions, contouring to improve force distribution, and adjusting trimlines to achieve suspension while preserving range of motion and ease of donning [3,9,10]. Because these modifications rely on tacit clinical knowledge and patient-specific considerations, rectification remains one of the most difficult stages to formalize and standardize within digital workflows. Furthermore, clinicians often report difficulty translating these hands-on, tactile rectification skills into software-based environments [10,11].

To address these challenges, researchers have proposed algorithmic approaches to partially automate digital rectification. For instance, optimization-based workflows have used finite element analysis and surrogate modeling to estimate socket mechanics [12], and then used multi-objective genetic algorithms to generate candidate transtibial socket designs [13]. Other studies have used sensor-based rule systems to propose rectification actions and directly modify 3D models [14], and statistical-shape models have also been used to generate evidence-based sockets from prior records for lower-limb applications [15]. While most studies have focused on lower-limb sockets, more recent work has extended evidence-based strategies to upper-limb applications, including approaches that generate multiple candidate sockets based on prior prosthetist designs [16].

Many of these data-driven approaches aim to encode expert practice into quantitative design rules. Therefore, to inform such methods, researchers have also focused on characterizing and quantifying socket shape and rectification practice directly, particularly in lower-limb applications [3,7,9,17]. However, the findings of these studies cannot be directly transferred to upper-limb applications, including transradial prostheses, which have distinct design requirements. Similar quantitative research on transradial socket design is relatively sparse; notably, only one study has characterized the rectification patterns of prosthetists designing Northwestern-style three-quarter sockets, providing an important foundation for data-driven upper-limb socket design [10].

While quantifying rectification practice for transradial sockets is an important step, prior work has primarily characterized shape changes from the captured cast model to the rectified cast model, rather than from the residual limb to the socket itself. This distinction is important because the starting shape for rectification is often not identical to the residual-limb shape; the casting process itself can introduce clinically meaningful shape variations through selective soft-tissue compression and posture correction during shape capture [18]. Consequently, rectification patterns alone do not fully explain how the shape of the residual limb is transformed into that of a final well-fitting socket. To our knowledge, no prior study has directly characterized the relationship between the geometry (i.e., the quantitative, measurable properties of the shape) of the residual limb and final socket. To address this gap, this study provides a preliminary characterization of this relationship using linear regression modeling to identify simple, interpretable, and clinically relevant limb–socket relationships.

## 2. Methods

### 2.1. Ethics statement

This study was approved by the Research Ethics Board at Holland Bloorview Kids Rehabilitation Hospital (File #19–864; approved 21 January 2020). Written informed consent was obtained from all adult participants. For participants under the age of 18 years, written informed consent was obtained from a parent or legal guardian, and written assent was obtained from the participant.

### 2.2. Data collection

A convenience sample of fourteen (n = 14) participants with transradial limb absence was recruited for this study between March 2023 and January 2025, and is consistent with prior research of similar scope [10,16,19]. The inclusion criteria were as follows: a) participants must have had transradial limb absence for a minimum of two years, and b) a new prosthetic socket was to be designed during the study period following shape capture. Table 1 lists the participant demographics, where each participant contributed a single limb–socket pair to the analysis, with no participant appearing more than once in the dataset.

**Table 1:** Participant demographics.

Participant	Age	Sex	Qualitative descriptors	
			Shape*	Length**
**P01**	47	M	Conical	Short
**P02**	10	F	Cylindrical	Short
**P03**	29	F	Conical	Short
**P04**	52	M	Conical	Short
**P05**	22	F	Conical	Short
**P06**	10	M	Cylindrical	Short
**P07**	29	F	Cylindrical	Short
**P08**	7	F	Conical	Long
**P09**	32	F	Cylindrical	Short
**P10**	24	M	Conical	Long
**P11**	13	F	Cylindrical	Very Short
**P12**	22	F	Conical	Very Short
**P13**	15	F	Conical	Short
**P14**	51	M	Cylindrical	Very Short

*Defined in accordance with ISO 8548–3:2025 – Prosthetics and Orthotics – Limb Deficiencies – Part 3: Method of Describing the Residual Limb after Upper Limb Amputation [20]; categorized as “Cylindrical”, “Conical”, or “Bulbous”.**Defined in accordance with Principles of Amputation, Physiopedia [21]; categorized as “Long”, “Short”, or “Very Short”.

The Spectra scanner (resolution: 0.1 mm) from Vorum (Vancouver, Canada) was used to digitize 3D models of participants’ residual limbs and sockets. Prior to scanning, the prosthetist clearly marked anatomical landmarks, including the epicondyles and olecranon, with indelible ink. Next, a 3D-printed fiducial marker (Fig 1a) was secured to the proximal upper arm (biceps region), positioned away from the region of interest (i.e., outside the region of contact of the socket), to facilitate subsequent alignment of models. The limb and fiducial marker were covered with a white nylon sock to improve scan quality by ensuring color homogeneity. The participant then donned the socket (Fig 1b), and three scans were acquired using an established technique [4]:

**Fig 1:**
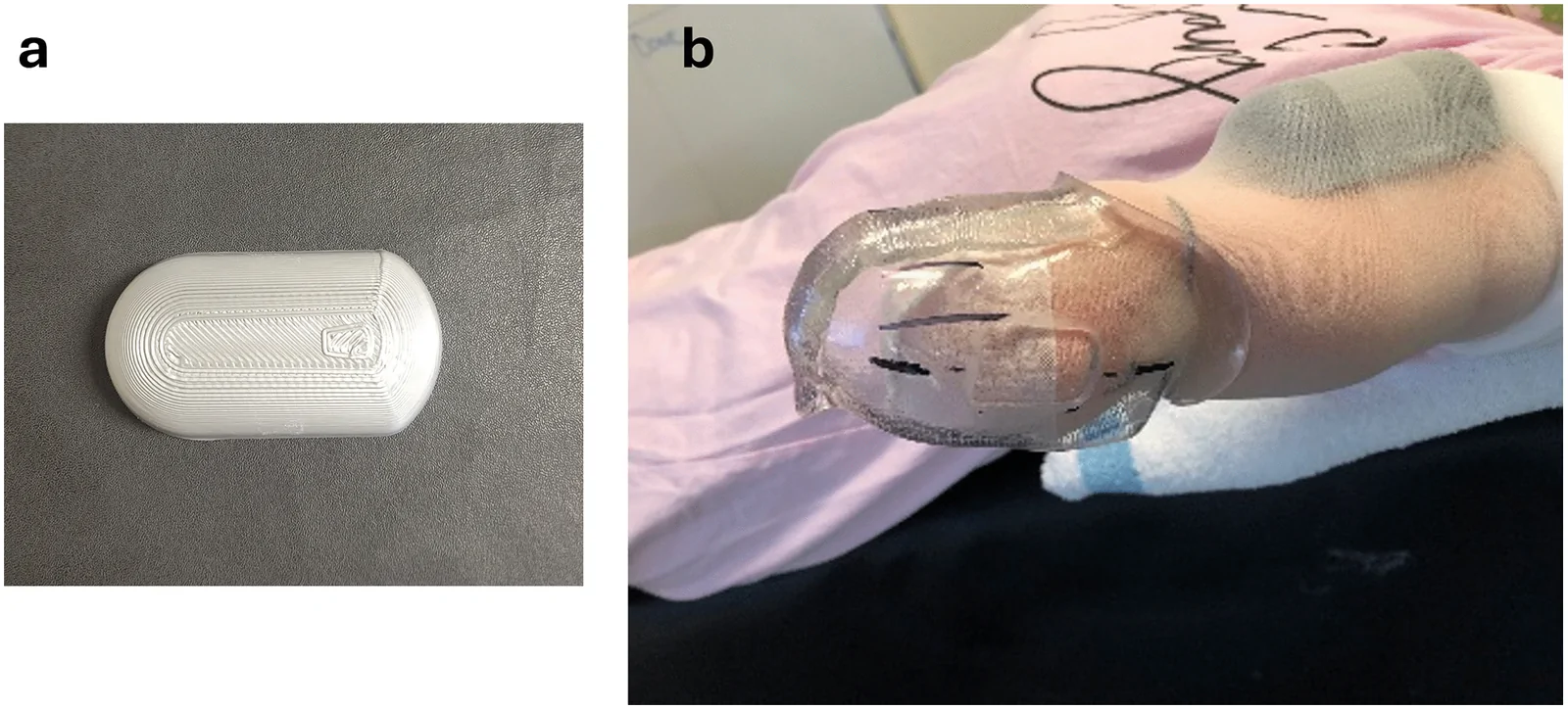
Scan preparation for limb–socket registration: images of the a) 3D-printed fiducial marker and b) limb with the fiducial marker attached, socket donned, and nylon sock worn.

1. **Limb–socket–fiducial (LSF)**: Residual limb with the fiducial marker attached and the socket donned (Fig 1b).
2. **Limb–fiducial (LF)**: Limb with the fiducial marker attached, without the socket (same setup as Fig 1b, excluding the socket).
3. **Socket (S)**: Positive mold of the cast of the socket created by the prosthetist.

### 2.3. Data preparation

All scans were post-processed in Canfit O&P and Spectra software (Vorum, Canada) to remove artifacts. Then, all limb models were manually aligned to a consistent anatomical coordinate system using a previously established landmark-based convention [4]. The coordinate axes were defined such that the x-, y-, and z-axes corresponded to the frontal, sagittal, and longitudinal axes, respectively (Fig 2). This preliminary step ensured consistent orientation across participants prior to limb–socket registration.

**Fig 2:**
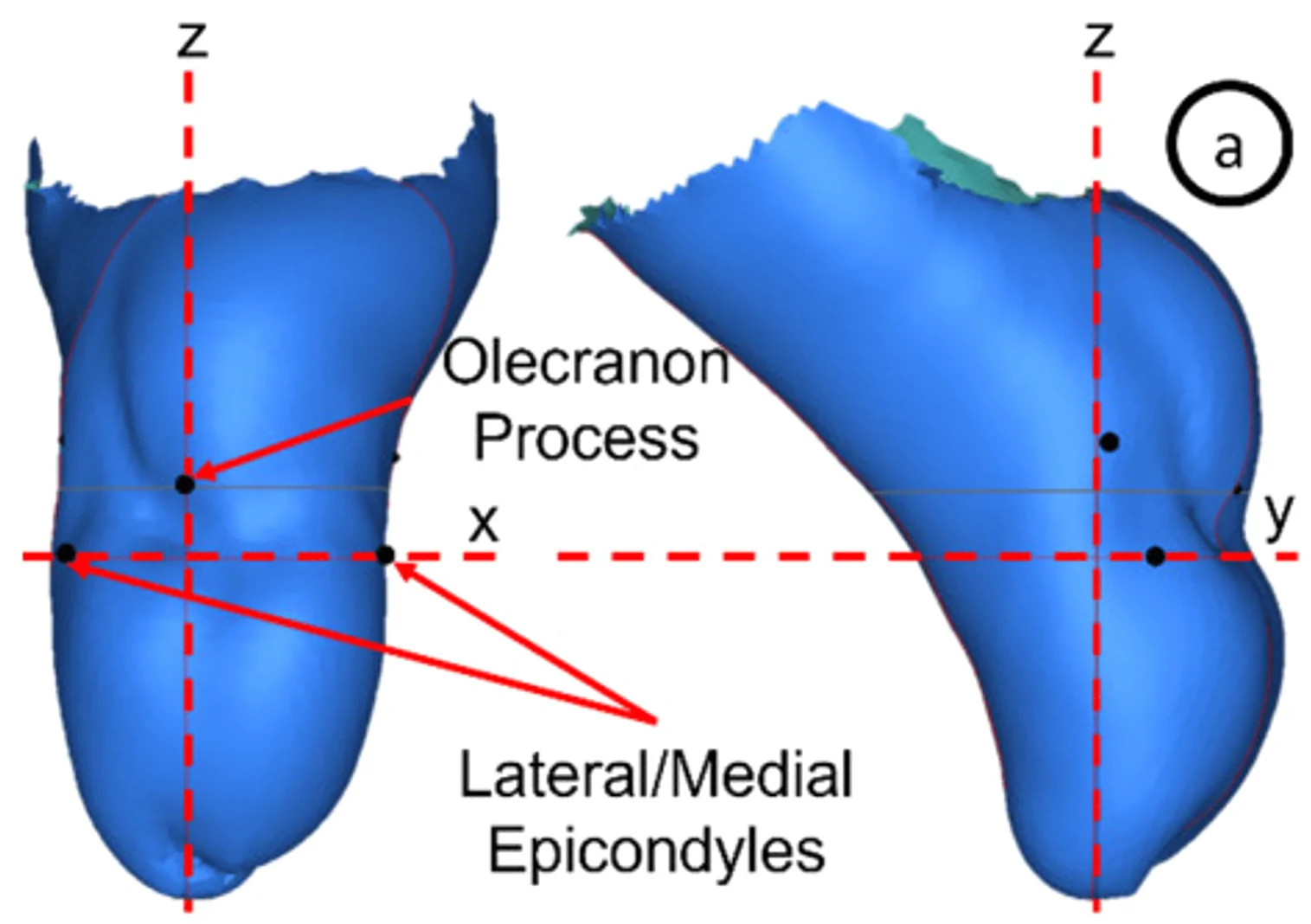
Landmark-based coordinate convention proposed by Ngan et al. [4], where the x-, y-, and z-axes correspond to the frontal, sagittal, and longitudinal axes, respectively.

### 2.4. Limb–socket alignment

To characterize limb–socket geometric relationships, each residual-limb model (**LF**) was registered to its corresponding socket model (**S**). Because the limb and socket differ significantly in shape, registration was performed in two stages: 1) the intermediate scan (**LSF**) was used to connect the two models by establishing a common reference frame, and 2) limb pose was corrected using a spline-based method adapted from a previous study [10].

#### 2.4.1. Establishing a common reference frame.

First, the socket model (**S**) was aligned to **LSF** through translational and rotational transforms using the visible socket trimlines as guides. Next, the limb-only scan (**LF**) was aligned to **LSF** using the fiducial marker as a reference, which was assumed to remain in a fixed position on the limb across scans.

With both models registered to **LSF**, **LF** is approximately aligned to **S**, providing an initial estimate of the limb’s position within the socket.

#### 2.4.2. Correcting limb pose.

Unlike the alignment for **S** and **LSF**, which depends on clear trimlines and rigid models, the alignment of **LF** to **LSF** is affected by small, clinically realistic posture differences between scan acquisitions (particularly at the elbow). Therefore, we corrected for elbow flexion using a reference-axis and curvature-based elbow detection method adapted from a previous study [10]. Specifically,

1. For each **LF** and **LSF**, a proximal–distal axis was computed using slicing, centroid computation, and Savitzky–Golay filtering (Fig 3a).
2. A semi-automated MATLAB (Mathworks Inc, CA, USA) script then identified the point of maximum curvature along the reference axis using the Frenet–Serret formulas. This point was treated as the elbow location, with the segments below and above the point corresponding to the forearm and upper arm, respectively (Fig 3b). The elbow flexion angles for LF and LSF were computed as $\theta_{LF}$ and $\theta_{LSF}$, respectively, and their difference was defined as:

**Fig 3:**
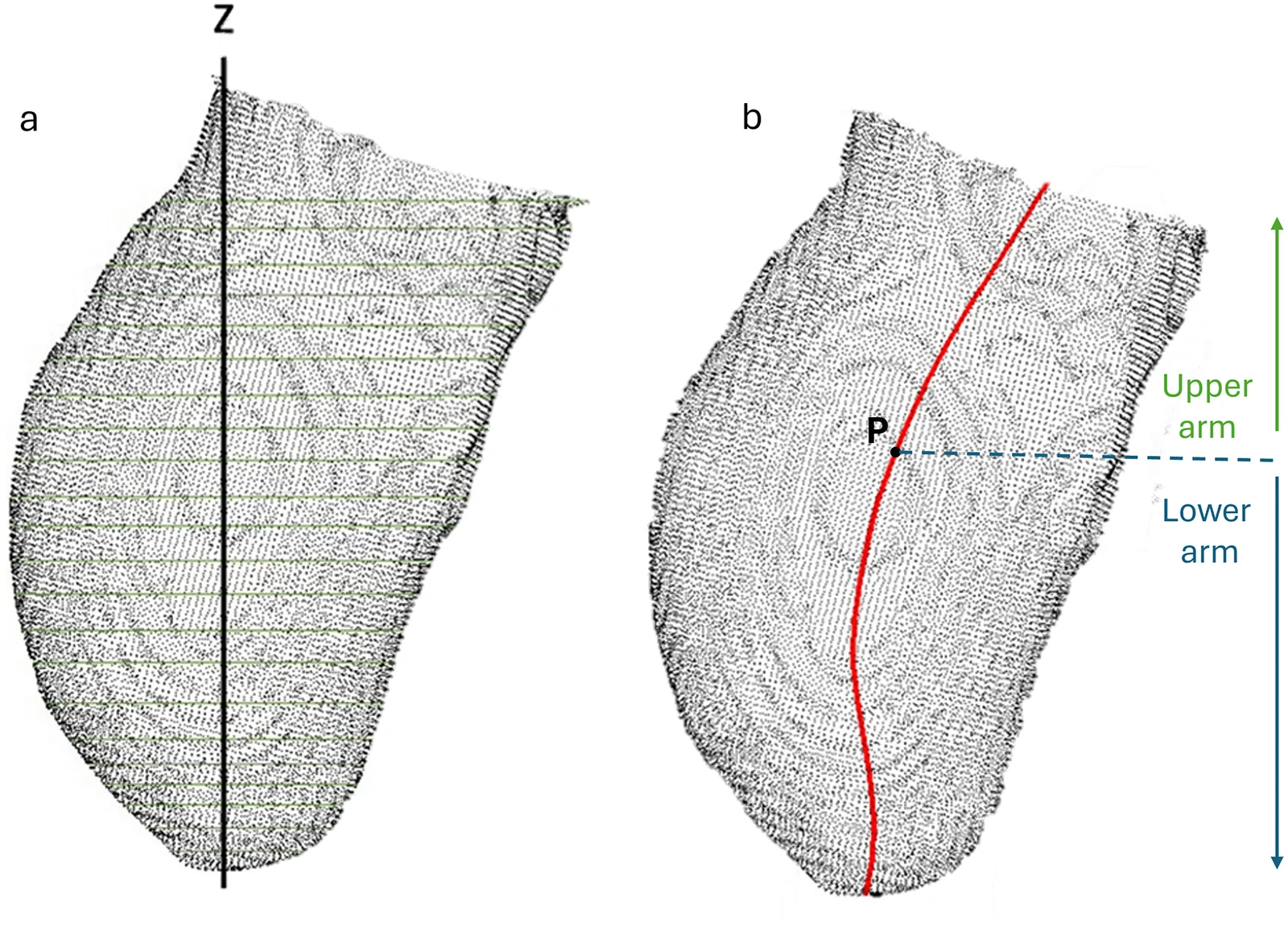
Reference-axis and curvature-based elbow detection for pose correction: a) Slicing the residual limb along the proximal–distal direction to compute centroids for subsequent smooth reference-axis computation; b) computing the point of maximum curvature (P) to detect the elbow location, separating the upper arm (green) from the lower arm (blue). Figure adapted from Ngan et al. (2024) [10]. The same process was repeated for the LSF model.


$$\begin{array}{c} \theta =\theta_{LF}-\theta_{LSF} \end{array}$$

The LF model was then rotated by θ in Canfit O&P, allowing the application of anatomically feasible rotation about the elbow joint while constraining any shape changes to the model in the socket contact region.

This alignment method yields a meaningful estimate of the residual-limb position within the socket, representative of the natural donned state. A flowchart of the entire limb–socket alignment process is shown in Fig 4.

**Fig 4:**
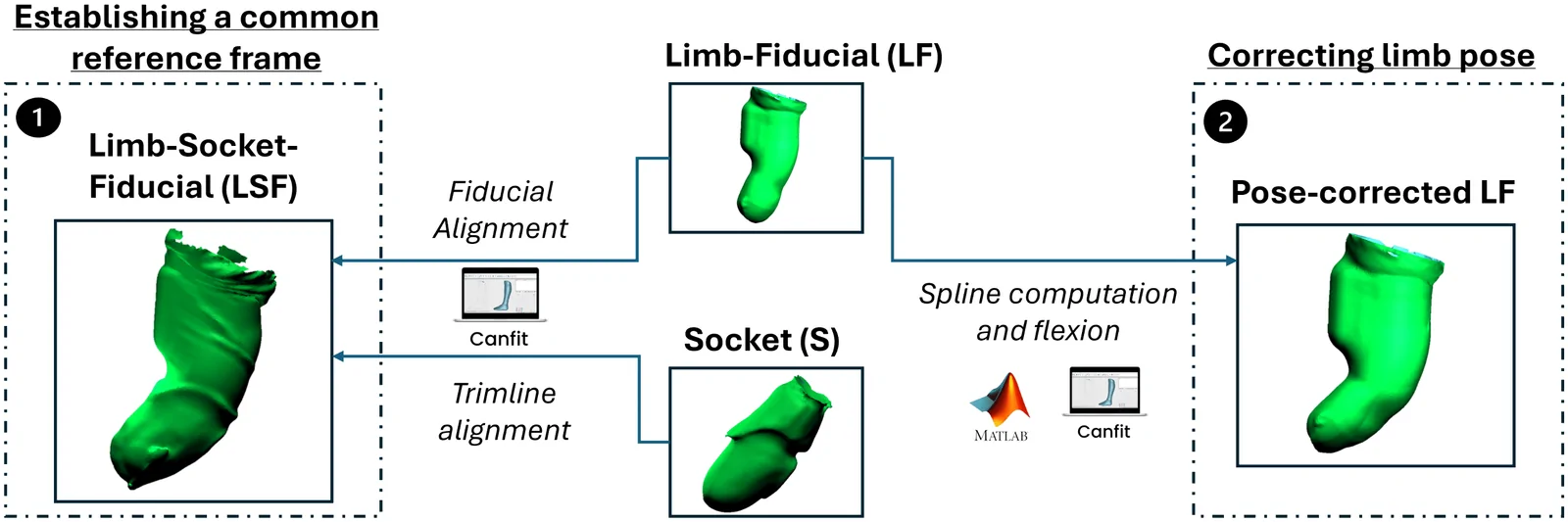
Flowchart for limb–socket alignment: 1) Aligning the limb (LF) and socket (S) using the limb–socket–fiducial (LSF) as a reference scan. 2) Correcting limb pose using the spline and curvature-based method to estimate elbow flexion differences.

### 2.5. Linear regression

To quantify the relationship between residual-limb geometry and corresponding socket geometry, we performed simple linear regressions using least squares on geometric descriptors for each limb–socket pair. For each descriptor, the socket value was treated as the dependent variable and the limb value as the independent variable. Linear regression was selected because this study aimed to identify simple, interpretable relationships between limb and socket descriptors. While multivariate and nonlinear approaches offer greater flexibility, the small sample size makes such models prone to overfitting, and the absence of prior quantitative data on transradial socket design makes it difficult to identify alternative model forms. Therefore, simple linear models represent a reasonable starting point that can be built upon as larger datasets become available. Such linear models can test whether the relationships are reasonably predictable while also preserving clinical interpretability [22,23].

The descriptors were selected based on prior prosthetics and orthotics literature to ensure clinical relevance, and intend to capture overall limb and socket dimensions, as well as local cross-sectional geometry. Global descriptors included volume [8,24], surface area [2,7], and proximal–distal length [12]. Cross-sectional descriptors were also included by slicing both the limb and socket models along the proximal–distal axis; separate regressions were fit at 25%, 50%, and 75% of the proximal–distal length (Fig 5) for the circumference [3], cross-sectional area [3,7,25], anterior–posterior (A–P) length [4], and mediolateral (M–L) length [26]. To facilitate the slicing process, the proximal end of both the limb and socket models was defined as the level of the proximal trimline, with the limb trimmed accordingly. Slice locations were then computed independently for each model, rather than at corresponding locations. This reflects the practical predictive use case, where the socket cannot be accurately aligned to the limb and slices must, therefore, be estimated independently.

**Fig 5:**
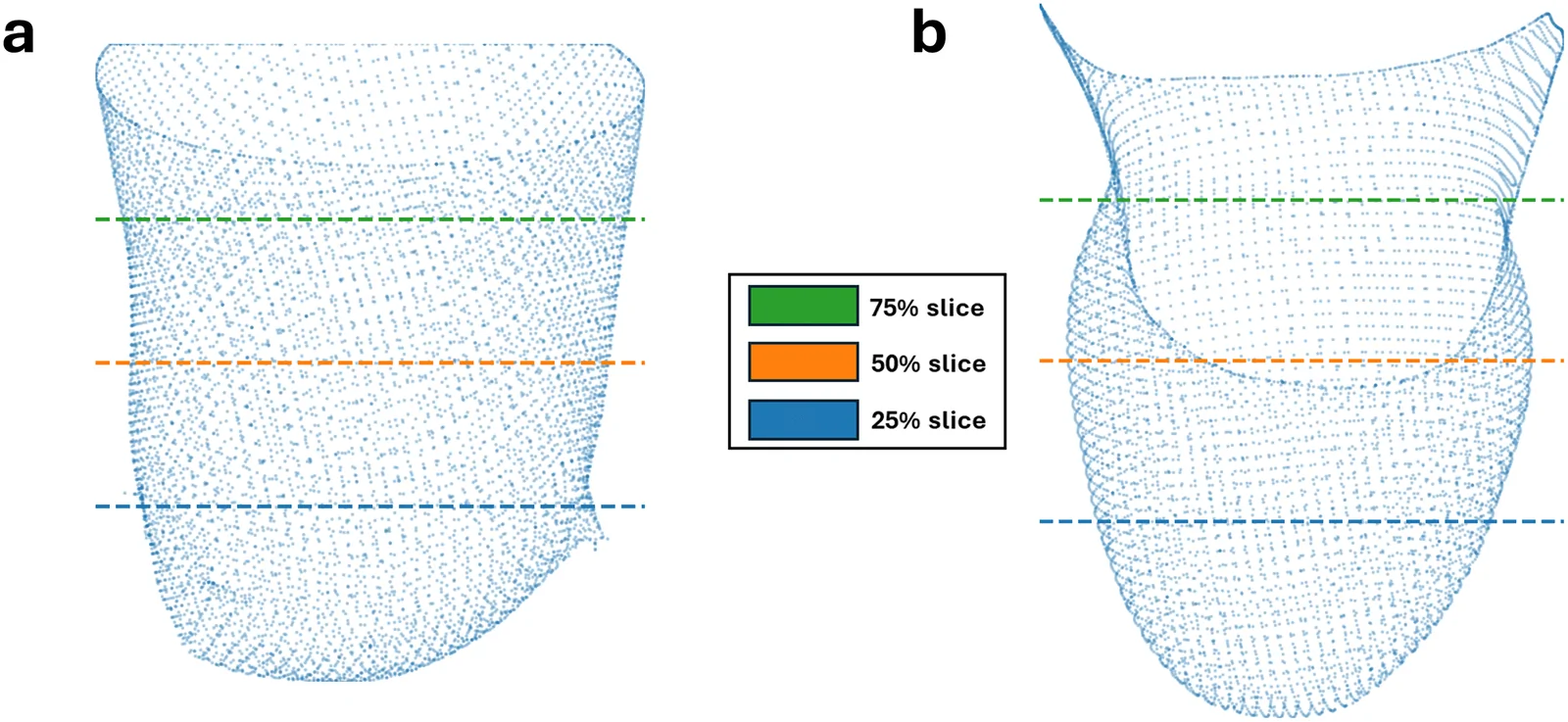
Frontal view of 25%, 50%, and 75% slice locations for the a) residual limb and b) socket. Slice-wise geometric descriptors were computed at these levels for each participant.

#### 2.5.1. Regression equations.

For each descriptor, we fit the following model:


$$\begin{array}{c} y=a\;+\;bx\;+\varepsilon \end{array}$$

where x is the limb descriptor value, y is the corresponding socket descriptor value, a is the intercept, b is the slope, and ε is the residual error. Model coefficients (*a, b)* were estimated using linear regression. The coefficient of determination (*R*^*2*^) was computed to summarize the explained variance, and the root-mean-square error (RMSE) was calculated to quantify the absolute prediction error.

For global descriptors, linearity is a reasonable assumption as overall socket size is expected to scale proportionally with limb size [16]. For cross-sectional descriptors, linearity is used as a starting point given the absence of prior quantitative data on these relationships, although local geometry is known to be affected by multiple interacting factors [10,27].

#### 2.5.2. Leave-one-out cross-validation (LOOCV).

To assess the generalizability of the per-attribute limb–socket regressions, we performed leave-one-out cross-validation (LOOCV) independently for each descriptor pair. In each LOOCV fold, one participant was held out as the test case, and a linear model was fitted on the remaining n − 1 participants. The fold-specific regression equation was then used to predict the held-out socket value $y_{pred}$ from the held-out limb value $x_{test}$:


$$\begin{array}{c} y_{pred}=\hat{a\;}+\;\hat{b}x_{test} \end{array}$$

where ($\hat{a\;},\hat{b\;}$) are the regression coefficients from the training set in that fold.

Prediction performance was determined using the predicted $R^{\text{2}}$ ($R_{pred}^{\text{2}}$) [28], which reflects how well the model predicts unseen participants. Additionally, we computed error metrics derived from the residual (${y_{true}-y}_{pred}$) [29–31], where $y_{true}$ is the actual observed socket value for the given descriptor:

1. **Mean absolute error (MAE)**, $\left|{y_{true}-y}_{pred}\right|$, which represents the typical magnitude of prediction error.
2. **Root-mean-square error (RMSE)**, $\sqrt{{\left({y_{true}-y}_{pred}\right)}^{\text{2}}}$, which also summarizes the prediction error but is more sensitive to outliers; consistency between the MAE and RMSE can be interpreted as indicative of stable error behavior, without large outliers.
3. **Mean percent error (MPE)**, $\text{1}\text{0}\text{0}\times \left(\frac{{y_{true}-y}_{pred}}{y_{true}}\right)$, which quantifies the signed percentage error and indicates systematic over- or under-prediction.
4. **Mean absolute percent error (MAPE)**, $\text{1}\text{0}\text{0}\times \left|\frac{{y_{true}-y}_{pred}}{y_{true}}\right|$, which provides an interpretable percentage-based measure of the error magnitude that normalizes for scale differences, enabling comparison across descriptors with different units.

These metrics were then averaged across all LOOCV folds to obtain per-metric performance summaries. Finally, since the sample size for this study is small, we calculated the retention to assess whether the model was overfit to the training data [32]. The retention was calculated as follows:


$$\begin{array}{c} Retention\;=\;\frac{R_{pred}^{\text{2}}}{R^{\text{2}}} \end{array}$$

Finally, to contextualize model performance, we examined participant-level MAPE values relative to commonly cited prosthetic-fit thresholds of 5% and 10% [8,33]. In the absence of established benchmarks for transradial sockets, these thresholds from lower-limb literature are used as a general reference frame for interpreting prediction accuracy. For each descriptor, we calculated the proportion of participants whose absolute prediction errors were within each threshold.

### 2.6. Sensitivity analysis

While the limb–socket alignment was reviewed independently by two prosthetists, small registration errors are inevitable. Therefore, we conducted a sensitivity analysis to evaluate the robustness of the spline-based pose-correction algorithm. Since global descriptors are unaffected by alignment, only slice-wise descriptors were assessed.

Alignment error was simulated by applying small rigid-body rotations about the centroid of each aligned limb model, with the socket model held fixed. Rotational perturbations of ±2°, ± 4°, and ±6° were applied about each of the three anatomical axes (18 perturbation conditions in total), consistent with clinical expectations and prior alignment studies [34,35]. Rigid-body rotation was used as a proxy for registration errors most likely to affect the workflow, including landmark placement and elbow-detection error. For each perturbed model, the slice-wise descriptors were recomputed and the corresponding regressions refitted. The resulting change in the fitted slope (|Δb|) and coefficient of determination (|Δ$R^{\text{2}}$|) relative to the unperturbed models was quantified to assess the effect of alignment error on the regression results.

## 3. Results

### 3.1. Regression equations

Linear regression models were fit for each limb–socket geometric descriptor (n = 14). Fig 6 presents the scatter plots and fitted regression lines with shaded 95% confidence interval bands for both global (Fig 6a–c) and cross-sectional descriptors (Fig 6d–g).

**Fig 6:**
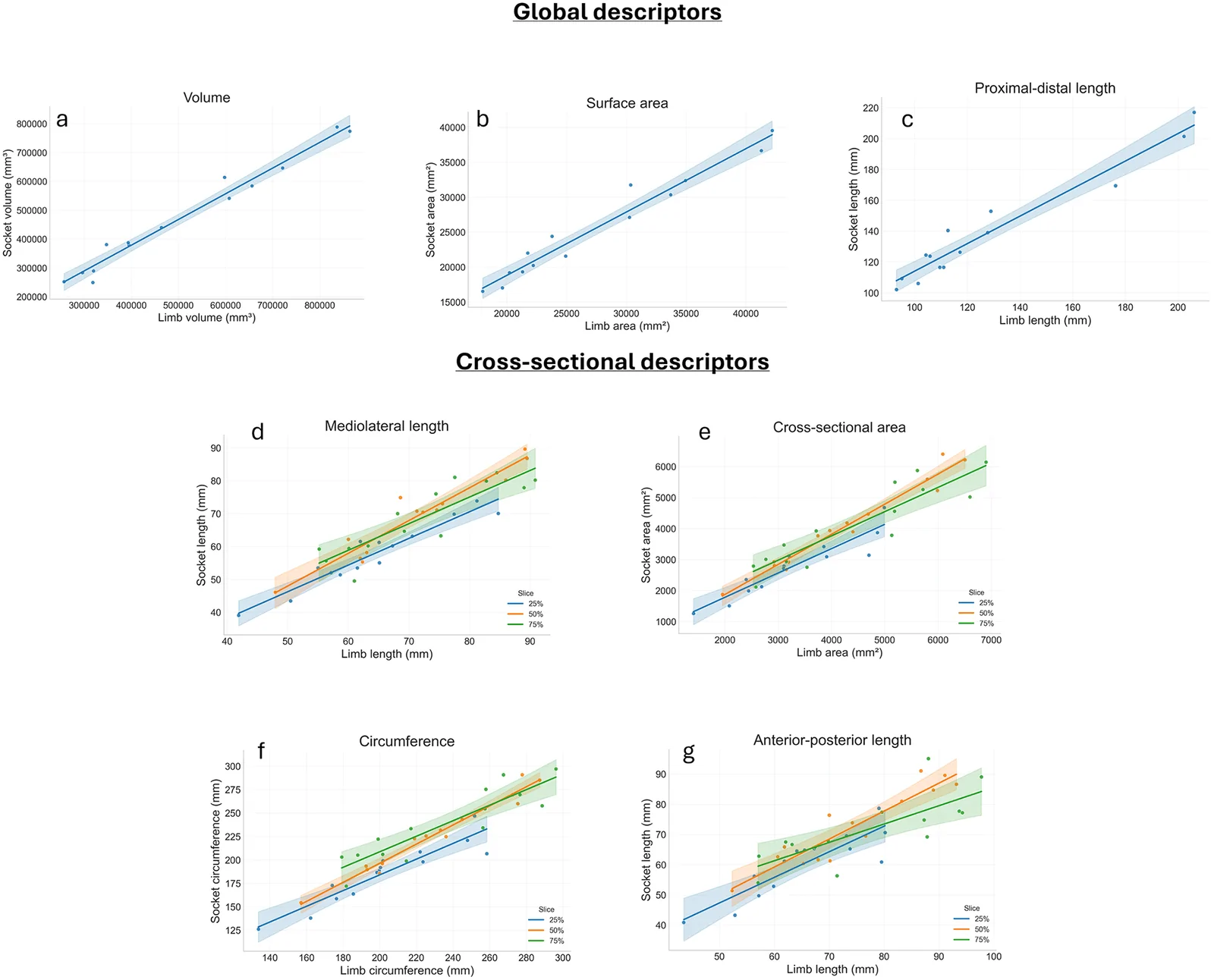
Limb-to-socket linear regression results for all geometric descriptors (n = 14). Global descriptors: (a) volume, (b) surface area, and (c) proximal–distal length. Cross-sectional descriptors at 25%, 50%, and 75% of proximal–distal length: (d) mediolateral (M–L) length, (e) cross-sectional area, (f) circumference, and (g) anterior–posterior (A–P) length. Points denote participant data, solid lines denote fitted regression lines, and shaded regions denote 95% confidence bands.

Table 2 reports the regression coefficients, 95% confidence intervals, and both goodness-of-fit metrics (R² and RMSE). For **global descriptors**, the regressions exhibited explained variances of $R^{\text{2}}=\text{0}.\text{9}\text{4}-\text{0}.\text{9}\text{7}\;$across all three descriptors. The fitted slopes were b=0.89−0.91, and the RMSE values were 8.1 mm, 1467.5 mm^2^, and 29056 mm^3^ for proximal–distal length, surface area, and volume, respectively.

**Table 2:** Ordinary least squares regression equations relating socket descriptors (dependent variable) to limb descriptors (independent variable), along with relevant goodness-of-fit metrics.

*Panel*	Descriptor	Slice	Intercept a (SE^*^)	Slope b (SE)	95%CI for b	R²	RMSE
***a***	Volume (mm³)	Global	21787.2 (22916.9)	0.89 (0.04)	[0.80, 0.98]	0.97	29056.0
***b***	Surface area (mm²)	Global	643.8 (1560.9)	0.91 (0.06)	[0.79, 1.03]	0.96	1467.5
***c***	Proximal-distal length (mm)	Global	24.7 (8.5)	0.89 (0.06)	[0.75, 1.03]	0.94	8.1
***d***	Mediolateral length (mm)	25%	5.7 (4.6)	0.81 (0.07)	[0.66, 0.96]	0.92	2.8
		50%	-1.8 (5.9)	1.00 (0.08)	[0.82, 1.17]	0.92	3.4
		75%	10.3 (9.3)	0.81 (0.13)	[0.53, 1.09]	0.77	5.1
***e***	Cross-sectional area (mm²)	25%	209.0 (308.5)	0.79 (0.09)	[0.59, 0.98]	0.87	322.0
		50%	-74.1 (241.1)	0.97 (0.05)	[0.85, 1.09]	0.96	252.5
		75%	630.0 (483.9)	0.78 (0.10)	[0.56, 1.01]	0.83	527.7
***f***	Circumference (mm)	25%	16.3 (19.9)	0.84 (0.10)	[0.63, 1.05]	0.86	11.5
		50%	-7.5 (12.5)	1.02 (0.05)	[0.90, 1.14]	0.97	7.0
		75%	42.9 (27.7)	0.83 (0.12)	[0.57, 1.08]	0.81	16.1
***g***	Anterior-posterior length (mm)	25%	5.0 (8.9)	0.85 (0.14)	[0.55, 1.14]	0.77	4.9
		50%	3.7 (8.0)	0.93 (0.11)	[0.69, 1.16]	0.86	4.5
		75%	24.9 (11.2)	0.61 (0.14)	[0.30, 0.92]	0.60	6.9

*SE denotes the standard error.

For **cross-sectional descriptors**, model fit varied by descriptor and slice location. Across all slice levels, slopes ranged from 0.61 to 1.02, and $R^{\text{2}}$ ranged from 0.60 to 0.97. The best fits were observed at the 50% slice for cross-sectional area and circumference ($R^{\text{2}}=\text{0}.\text{9}\text{6}$ and 0.97), while the lowest fit was found for A–P length at the 75% slice ($R^{\text{2}}=\text{0}.\text{6}\text{0}$). Furthermore, across all descriptors and slices, the RMSE ranged from 2.8–5.1 mm (M-L length), 4.5–6.9 mm (A-P length), 7.0-16.1 mm (circumference), and 252.5–527.7 mm^2^ (area). In terms of slice locations, the fit was highest at the 50% slice (average $R^{\text{2}}=\text{0}.\text{9}\text{3}$), followed by the 25% slice (average $R^{\text{2}}=\text{0}.\text{8}\text{5}$), and finally the 75% slice (average $R^{\text{2}}=\text{0}.\text{7}\text{5}$).

### 3.2. LOOCV results

Table 3 summarizes the LOOCV performance for each limb–socket descriptor. Across all descriptors and slice locations, the $R_{pred}^{\text{2}}$ ranged from 0.48 to 0.97, with an overall mean (SD) of 0.83 (0.13). Further, the MAPE ranged from 2.5%–13.6%, while the MPE ranged from -2.3%–0.0%.

**Table 3:** Leave-one-out cross-validation (LOOCV) performance of limb–socket linear regressions. The predicted R^2^ and error metrics are reported for global and slice-based descriptors. 95% Confidence intervals are reported in square brackets.

*Panel*	Descriptor	Slice	R^2^_pred_	MAE	RMSE*	MPE (%)	MAPE (%)
***a***	Volume (mm³)	Global	0.97	26048.4	33311.8 [14760.8, 37336.0]	-0.9 [-5.8, 4.0]	6.2 [2.5, 9.8]
***b***	Surface area (mm²)	Global	0.95	1349.2	1663.1 [820.6, 1877.8]	-0.6 [-4.1, 2.9]	5.4 [3.5, 7.3]
***c***	Proximal-distal length (mm)	Global	0.92	8.1	9.6 [5.4, 10.9]	-0.5 [-4.1, 3.2]	5.8 [4.0, 7.6]
***d***	Mediolateral length (mm)	25%	0.88	2.6	3.3 [1.6, 3.7]	-0.5 [-3.5, 2.5]	4.6 [2.8, 6.3]
		50%	0.90	2.8	3.8 [1.5, 4.2]	-0.3 [-3.4, 2.8]	4.2 [2.2, 6.3]
		75%	0.70	4.9	5.8 [3.1, 6.6]	-0.8 [-5.8, 4.2]	7.3 [4.1, 10.4]
***e***	Cross-sectional area (mm²)	25%	0.79	313.2	397.7 [180.0, 446.4]	-2.1 [-9.5, 5.4]	11.4 [7.1, 15.7]
		50%	0.95	217.2	303.0 [102.4, 332.1]	-0.2 [-3.9, 3.5]	5.4 [3.1, 7.7]
		75%	0.77	514.5	609.7 [336.8, 692.3]	-2.3 [-11.1, 6.4]	13.6 [8.8, 18.5]
***f***	Circumference (mm)	25%	0.79	11.3	14.4 [6.4, 16.1]	-0.7 [-4.7, 3.3]	6.0 [3.6, 8.4]
		50%	0.96	5.8	8.2 [2.6, 9.0]	0.0 [-1.9, 1.8]	2.5 [1.3, 3.8]
		75%	0.74	16.2	18.8 [11.0, 21.3]	-0.6 [-5.1, 3.9]	7.0 [4.7, 9.4]
***g***	Anterior-posterior length (mm)	25%	0.67	5.0	5.9 [3.2, 6.7]	-1.2 [-6.6, 4.2]	8.4 [5.4, 11.4]
		50%	0.83	4.3	5.1 [2.8, 5.8]	-0.4 [-4.3, 3.5]	6.1 [4.0, 8.1]
		75%	0.48	6.4	7.9 [3.8, 8.9]	-1.0 [-6.9, 4.9]	8.9 [5.5, 12.4]
	**Average across sample (SD)**		**0.83 (0.13)**			**-0.8 (9.0)**	**6.9 (5.9)**

*95% CIs are identical for MAE and RMSE.

For **global descriptors**, LOOCV performance remained high, with $R_{pred}^{\text{2}}$ values of 0.97, 0.95, and 0.92 for volume, surface area, and proximal–distal length. The mean MAPE was 5.8% (4.8) and the mean MPE was -0.7%. For **cross-sectional descriptors**, LOOCV results varied by descriptor and slice location. The mean $R_{pred}^{\text{2}}$ values were 0.84 for cross-sectional area, 0.83 for M–L length, 0.83 for circumference, and 0.66 for A–P length. Across slice locations, the mean $R_{pred}^{\text{2}}$ values were 0.91 at the 50% slice, 0.78 at the 25% slice, and 0.67 at the 75% slice. For all cross-sectional descriptors, the mean MAPE was 7.1% (6.1) and the mean MPE was -0.8%.

Finally, the retention values were calculated to assess overfitting and are reported in Table 4. The global mean retention is 0.99; the slice-wise mean retentions are 0.92, 0.98, and 0.89 at the 25%, 50%, and 75% levels, respectively (overall slice-wise mean: 0.93).

**Table 4:** Retention values for all descriptors to assess overfitting.

	Volume	Surface area	P–D length	M–L Length			CSA			Circumference			A–P Length		
				25%	50%	75%	25%	50%	75%	25%	50%	75%	25%	50%	75%
**Retention**	0.99	0.99	0.98	0.96	0.97	0.91	0.91	0.99	0.93	0.92	0.99	0.92	0.87	0.96	0.80

Finally, Table 5 shows the percentage of participants whose LOOCV prediction errors fell within 5% and 10% thresholds for each descriptor, providing a participant-level view of threshold attainment across global and slice-wise measures.

**Table 5:** Percentage of participants falling within fit thresholds established by prior prosthetics literature [24,33,36].

Criterion	Volume	Surface area	P–D length	M–L Length at 25, 50, and 75% locations			CSA at 25, 50, and 75% locations			Circumference at 25, 50, and 75% locations			A–P Length at 25, 50, and 75% locations		
				25	50	75	25	50	75	25	50	75	25	50	75
**5%**	71%	57%	36%	64%	57%	36%	7%	57%	21%	57%	79%	43%	29%	43%	21%
**10%**	79%	86%	90%	100%	86%	79%	57%	79%	43%	79%	100%	71%	79%	86%	71%

### 3.3. Sensitivity analysis

Table 6 reports the change in the fitted slope and coefficient of determination for each slice-wise descriptor, averaged across all 18 perturbation conditions.

**Table 6:** Sensitivity of the slice-wise regressions to rotational perturbations of the limb model.

*Slice*	Descriptor	Slope	|Δb|	*R²*	|Δ*R²*|
***25%***	M–L length	0.81	0.019 (0.020)	0.92	0.011 (0.010)
	A–P length	0.85	0.014 (0.011)	0.77	0.011 (0.007)
	Circumference	0.84	0.016 (0.018)	0.86	0.010 (0.013)
	Cross-sectional area	0.79	0.011 (0.014)	0.86	0.008 (0.010)
***50%***	M–L length	1.00	0.009 (0.008)	0.92	0.007 (0.007)
	A–P length	0.93	0.014 (0.013)	0.86	0.007 (0.007)
	Circumference	1.02	0.007 (0.008)	0.97	0.001 (0.002)
	Cross-sectional area	0.97	0.008 (0.010)	0.96	0.002 (0.002)
***75%***	M–L length	0.81	0.015 (0.012)	0.77	0.014 (0.009)
	A–P length	0.61	0.019 (0.015)	0.60	0.036 (0.042)
	Circumference	0.83	0.006 (0.007)	0.81	0.009 (0.014)
	Cross-sectional area	0.78	0.005 (0.007)	0.82	0.008 (0.010)

Across all descriptors and conditions, the slopes changed by 0.012 and $R^{\text{2}}$ by 0.010 on average. The smallest changes occurred at the 50% slice and the largest at the 75% slice, with the maximum change in $R^{\text{2}}\;$observed for A–P length at the 75% slice.

## 4. Discussion

In recent years, healthcare systems have begun to adopt more standardized, data-driven methods to improve patient outcomes, reduce clinical burden, and expand access to care [37]. In prosthetics, this shift has focused on two related goals: 1) quantifying conventional socket-design practices to formalize clinician expertise [10,17] and 2) implementing digital tools, such as 3D scanning [4,38], computer-aided socket design [39], and additive manufacturing [40,41], to streamline socket design and fabrication.

Despite these advances, digital workflows also introduce some challenges, including the loss of tactile feedback and limited translation of clinician experience into software environments [10]. To address these limitations, several data-driven and algorithmic approaches have been developed to predict prosthetic socket shapes and characteristics [12,13,16]. However, because socket design is influenced by multiple factors, including residual-limb shape, age, and tissue consistency, no single predictive model is likely to be sufficient in all cases [7]. Instead, a more realistic goal may be to combine multiple complementary models, including those that generate a spectrum of candidate socket designs that represent the variations between established design approaches [13,16].

Within this context, the regression equations presented in this study should be interpreted as preliminary and exploratory quantitative descriptors of limb–socket scaling relationships in transradial sockets, intended to characterize general geometric trends in socket design rather than to be directly used as clinically deployable predictive tools. Specifically, they can be used to augment existing predictive models, helping such models to identify which candidate sockets align most closely with established limb–socket design trends, thereby supporting the selection of the most appropriate option for a given user. More broadly, these findings could both support prosthetists’ clinical intuition and contribute to greater standardization of socket design practice.

### 4.1. Regression equations

For the global descriptors (volume, surface area, and proximal–distal length) of all 14 limb–socket pairs, the regression models showed consistently high explained variance ($R^{\text{2}}$ = 0.94–0.97), with slopes near one (b = 0.89–0.91). In the field of prosthetics, while no specific studies exist on regressions between the limb and the socket, acceptable $R^{\text{2}}$ values for regressions between right and left limb measurements [42], intrasocket pressures–force relationships [43], and residual limb growth [44] were found to be 0.99, 0.93, and 0.74, respectively. Furthermore, prior research has shown that, for complex and multifactorial clinical relationships, a low $R^{\text{2}}$ value of 0.15–0.20 is still informative [45] – the relationship between the limb and socket shape, especially in localized regions, is highly multifactorial and dependent on not only geometric factors, but also soft-tissue characteristics, client demographics, and functional requirements [27].

Practically, our high $R^{\text{2}}\;$values for global descriptors indicate that most of the between-participant variation in socket size is captured by simple linear scaling with limb size (i.e., participants with larger limbs systematically receive larger sockets). The slight bias towards non-unity slopes and non-zero intercepts (Table 2) indicates that socket design reflects a combination of the aforementioned linear scaling and some systematic design offsets, differences among prosthetists’ design choices, and errors within the process. For instance, the intercept for the proximal–distal length (24.7 mm) indicates that there is a systematic positive offset in the socket for distal-end clearance, with some potential effects of local anatomy, specific design strategy, and soft-tissue characteristics [27]. It should be noted that these systematic offsets may partly reflect design conventions specific to the participating clinicians and clinic, such as the preferred amount of distal clearance, the degree of soft-tissue compression, or trimline placement, rather than universal socket-design principles. Studies across multiple clinicians and settings are critical to determining the generalizability of these relationships.

The slice-wise regressions showed that predictability was not uniform along the length of the socket. Cross-sectional relationships were strongest mid-length (50% slice: mean $R^{\text{2}}$ = 0.93), weaker in the distal third (25% slice: mean $R^{\text{2}}$ = 0.85), and lowest in the proximal third (75% slice: mean $R^{\text{2}}$ = 0.75). This is likely because the middle third is a region of the socket that is more directly governed by the overall bulk and shape of the residual limb [10]. In contrast, the proximal third of the limb and socket differ significantly in shape due to the presence of trimlines. Prosthetists also often make significant compensations above the epicondyles to achieve suspension, further exacerbating these differences [46,47]. Finally, the distal third is affected by the distal-end clearance and tapering, where small absolute differences can correspond to larger relative errors [10,27], collectively suggesting that a slice-wise linear model is less capable of capturing relationships at the proximal and distal ends of the socket, where design intent begins to dominate over limb shape. These regions are functionally important: the proximal region governs suspension and ease of donning, while the distal region accommodates limb growth and prevents excessive pressure on soft tissue [27].

While global regression models can estimate overall socket scale to inform predictive design workflows, the localized geometry in the proximal and distal regions has a greater influence on fit and comfort. Therefore, clinical expertise is a crucial complement to geometric modelling in these regions, where design decisions reflect patient-specific considerations and clinician choices that simple scaling relationships cannot capture [10,48].

### 4.2. LOOCV results

As reflected in Table 3, the LOOCV results confirm strong generalizability for global descriptors (mean $R_{pred}^{\text{2}}$ = 0.95), with weaker yet informative performance in localized slice-wise metrics (mean $R_{pred}^{\text{2}}$ = 0.79; by slice: 25% = 0.78, 50% = 0.91, 75% = 0.67). While reporting for LOOCV $R_{pred}^{\text{2}}$ for linear regression modeling in prosthetics is rare, adjacent fields like biology and chemistry often consider $R_{pred}^{\text{2}}$>0.5 to be strongly indicative of predictive ability [46,47]. In biomechanics, some kinematics research has reported $R_{pred}^{\text{2}}$ ranging between 0.82 and 0.93 [49,50], and a study on anthropometrics has reported a value of 0.94–0.98 [51]. Our $R_{pred}^{\text{2}}$ results lie within these ranges, reinforcing the idea that global socket dimensions are readily predictable from global limb size, while localized regions require richer modeling. Further, across descriptors, MPE values were close to zero (–2.3% to 0.0%), indicating minimal systematic bias and a slight tendency toward under-predicting socket dimensions compared to those designed by a prosthetist.

Because $R_{pred}^{\text{2}}$ is generally more conservative than in-sample $R^{\text{2}}$, we also examined retention to assess the extent to which model performance was maintained under cross-validation. The retention values in Table 4 were generally high (>0.90 for most descriptors), suggesting limited overfitting despite our small sample size [52,53]. However, small sample sizes can introduce some volatility in the coefficients and instability in model predictions, meaning that the reported slopes and intercepts could shift if the model were developed on a different sample of the same size [54]. While the LOOCV results suggest that the models maintain reasonable performance on unseen participants, the regression equations presented herein should still be interpreted as preliminary estimates pending validation in larger, more diverse cohorts.

To contextualize these predictive errors, we compared them to commonly cited socket-fit tolerance benchmarks, while recognizing that criteria for a well-fitting socket remain ill-defined [55]. In lower-limb applications, Fernie and Holliday classified a residual-limb volume change of 5% as good fit, 5–10% as acceptable fit, and >10% suggesting the need for a new socket [33]. Sanders et al. similarly suggested that an approximate 6% volume change indicates socket fit deterioration [8,24], while adaptive transtibial sockets have shown an acceptable within-session socket size range of 5.5% [36]. Because quantitative benchmarks for transradial sockets are not well-established, we use these lower-limb volume-based thresholds to interpret error percentages across descriptors with different units. The global MAPEs were relatively clustered (volume: 6.2% (6.9), surface area: 5.4% (3.7), and P–D length: 5.8% (3.5); mean global MAPE: 5.8%), suggesting that overall socket scale is predictably related to limb scale. In contrast, slice-wise errors were lowest at 50% (mean MAPE: 4.6%), higher at 25% (mean MAPE: 7.6%), and highest at 75% (mean MAPE: 9.2%), with the largest errors observed for cross-sectional area at the distal and proximal ends (11.4% and 13.6%, respectively), suggesting that shape differences due to trimline strategy and distal clearance make slice-level predictions difficult at the ends of the socket. It is important to note, however, that this does not imply an absence of structure in these regions; instead, it may suggest that their governing relationships are multivariate and incorporate factors such as trimline geometry, tissue consistency, and age [16,56]. For instance, pediatric participants generally require greater distal clearance to accommodate residual limb growth, meaning that age could contribute systematically to the variability observed at the distal end [16]. Similarly, soft-tissue mechanical properties vary with age [57]; for instance, tissue may become stiffer and less elastic in older individuals, which could affect the degree of compression applied during rectification and contribute to variability in localized geometry.

Since several mean prediction errors lay near commonly cited socket-fit thresholds, we examined the participant-level error distribution shown in Table 5 to determine whether these averages were driven by a small number of extreme cases. This pattern was descriptor-dependent: global descriptors remained within the 10% “acceptable” threshold for most participants, as did mid-socket metrics (50% slice). At the proximal and distal slices, the threshold attainment was lower, particularly for the cross-sectional area and circumference. Three participants showed notably larger errors: P06 (errors: 26.8%, 18.0%, and 12.5% at the 25% slice), P11 (errors: 31.0%, 23.7%, and 15.5% at the 75% slice), and P14 (errors: 30.5%, 23.5%, and 17.2% at the 25% slice). All three had cylindrical and short/very short residual limbs, suggesting that these shapes may be less well-represented by the current regression models. This indicates that future research should explore morphology-specific or subgroup-based models.

Finally, it is important to note that the regression models proposed herein are mainly intended to support algorithmic and data-driven socket workflows. While we have attempted to contextualize prediction errors against established fit thresholds to provide clinical interpretability, the absence of benchmarks for transradial sockets means that these comparisons should be interpreted with caution, and establishing such benchmarks remains an important direction for future research.

### 4.3. Sensitivity analysis

The regression estimates were largely insensitive to rotational perturbation of the limb model, with slopes changing by a mean of 0.012 and $R^{\text{2}}$ by 0.010 across all conditions (Table 6). The largest sensitivities were found for A–P length at the 75% slice (mean |Δ*R²*| = 0.036 and |Δb| = 0.019), which is also the descriptor with the weakest $R^{\text{2}}$ value, of 0.60. This finding is consistent with that of a previous study, which reported lower reliability for A–P than M–L measurements in transradial limb scanning and attributed this to small differences in elbow flexion between scans [4]. Across descriptors, the change in slope (|Δb|) due to alignment errors was small relative to the precision of the slope estimates themselves. The mean change in slope was only about 5.9% (SD: 2.7%) of the margin of error of the corresponding slope (i.e., half the width of its 95% confidence interval in Table 2), while |Δ*R²*| averaged 1.4% (SD: 1.5%) of the corresponding *R²* value in Table 2. This confirms that realistic alignment errors shift the regression estimates by only a fraction of their existing sampling uncertainty.

### 4.4. Clinical implications

In combination, the LOOCV results and regression equations indicate that the global scale of the socket is well-predicted by the global dimensions of the limb, while proximal and distal regions are more affected by trimline shape and distal-end clearance, making prediction difficult. These findings have several clinical implications:

- **Quantitative reference**: The presented regression equations can complement workflows that generate multiple candidate sockets [16] or scenarios where multiple predictive methods are combined to generate several candidates. In these contexts, they could potentially serve as a quantitative reference to flag designs that deviate from expected limb–socket relationships, helping to identify appropriate socket shapes from a range of options, although further validation in larger cohorts is necessary before such applications are realized.
- **Predictive modeling**: The relationships can provide preliminary and exploratory foundations for future work with larger sample sizes, and may serve as interpretable descriptors of limb–socket relationships that can inform future predictive modeling approaches.
- **Greater standardization**: By making some aspects of prosthetist design practice more quantitatively explicit, these findings could support greater consistency and transparency in socket design, particularly within newer digital methods.
- **Teaching tool**: These geometric trends identified in this study could contribute to prosthetist training by providing a preliminary quantitative basis for understanding which aspects of socket shape tend to scale predictably with limb geometry and those that are more strongly influenced by clinician judgment.

### 4.5. Limitations and future directions

This study has some key limitations that, if addressed in future research, could strengthen the generalizability and utility of these findings:

- **Sample size**: While consistent with previous studies of similar scope (n = 14), recruitment in upper-limb prosthetics research is inherently challenging because the population is relatively small. Therefore, the sample size in this study may not capture the full variability in the patient population, particularly because participants were recruited from a single hospital and treated by two clinicians: with only two clinicians contributing, clinician-level clustering could not be addressed through mixed-effects modeling [58]. Further, the regression relationships reported here may partially reflect local design practices or socket-specific conventions of the three-quarter Northwestern style design; future research should focus on larger, multi-clinic cohorts to improve representativeness and enable subgroup analyses.
- **Unmodeled descriptors**: Certain descriptors that could be important contributors to socket shape, including tissue consistency, distal clearance, and age, were not explicitly modeled, as the current regressions were limited to geometric descriptors. Age, in particular, could affect limb–socket relationships in several ways: pediatric users typically require greater distal clearance to accommodate growth, while differences in soft-tissue consistency between younger and older participants could affect rectification decisions and localized socket geometry. Future work should incorporate these variables as predictors for socket shape, since prior literature suggests that such patient- and design-specific factors can meaningfully influence socket geometry [16].
- **Model form**: While using single-predictor (univariate) linear regressions intentionally prioritizes clinical interpretability, this choice limits performance in regions where the shape is influenced by multiple interacting variables, e.g., the effects of age and soft tissue consistency at the distal end, in addition to local geometry. Future research should explore multivariate models, particularly in the proximal and distal thirds, where clinical intent dominates and richer representation is likely required.
- **Clinical error thresholds**: Clinically meaningful error thresholds for geometric descriptors are not clearly established in literature on prosthetic socket design, especially at the transradial level. The thresholds used here are borrowed from lower-limb literature and should be interpreted accordingly; future research should attempt to define descriptor-specific benchmarks, ideally in relation to clinical outcomes.

## 5. Conclusion

In this study, we quantified relationships between limb and socket shape through interpretable linear regression models across global and slice-wise geometric descriptors. Global descriptors exhibited strongly linear relationships and high generalizability under LOOCV, indicating that overall socket scale is largely predictable from limb size. In contrast, slice-wise descriptors were less predictable toward the proximal and distal ends, consistent with regions where socket geometry reflects targeted clinical design intent rather than proportional scaling. The findings provide an exploratory characterization of limb–socket geometric relationships that could, in future work, support data-driven socket design and guide future multivariate models incorporating additional patient- and design-specific factors.
